# Controlled spermatozoa–oocyte interaction improves embryo quality in sheep

**DOI:** 10.1038/s41598-021-02000-z

**Published:** 2021-11-19

**Authors:** Debora Agata Anzalone, Luca Palazzese, Marta Czernik, Annalaura Sabatucci, Luca Valbonetti, Emanuele Capra, Pasqualino Loi

**Affiliations:** 1 Faculty of Veterinary Medicine, University of Teramo, Campus Coste Sant’Agostino, Street R. Balzarini 1, 64100 Teramo, Italy; 2 Institute of Genetics and Animal Biotechnology of the Polish Academy of Sciences, 05-552 Jastrzebiec, Poland; 3 Faculty of Biosciences, and Technology for Food Agriculture and Environment, University of Teramo, 64100 Teramo, Italy; 4 Istituto di Biologia e Biotecnologia Agraria, Consiglio Nazionale delle Ricerche IBBA CNR, 26900 Lodi, Italy

**Keywords:** Embryology, Physiology

## Abstract

The current protocols of in vitro fertilization and culture in sheep rely on paradigms established more than 25 years ago, where Metaphase II oocytes are co-incubated with capacitated spermatozoa overnight. While this approach maximizes the number of fertilized oocytes, on the other side it exposes them to high concentration of reactive oxygen species (ROS) generated by active and degenerating spermatozoa, and positively correlates with polyspermy. Here we set up to precisely define the time frame during which spermatozoa effectively penetrates and fertilizes the oocyte, in order to drastically reduce spermatozoa-oocyte interaction. To do that, in vitro matured sheep oocytes co-incubated with spermatozoa in IVF medium were sampled every 30 min (start of incubation time 0) to verify the presence of a fertilizing spermatozoon. Having defined the fertilization time frame (4 h, data from 105 oocytes), we next compared the standard IVF procedures overnight (about 16 h spermatozoa/oocyte exposure, group o/nIVF) with a short one (4 h, group shIVF). A lower polyspermic fertilization (> 2PN) was detected in shIVF (6.5%) compared to o/nIVF (17.8%), *P* < 0.05. The o/nIVF group resulted in a significantly lower 2-cell stage embryos, than shIVF [34.6% (81/234) vs 50.6% (122/241) respectively, *P* < 0.001]. Likewise, the development to blastocyst stage confirmed a better quality [29% (70/241) vs 23.5% (55/234), shIVF vs o/nIVF respectively] and an increased Total Cell Number (TCN) in shIVF embryos, compared with o/n ones. The data on ROS have confirmed that its generation is IVF time-dependent, with high levels in the o/nIVF group. Overall, the data suggest that a shorter oocyte-spermatozoa incubation results in an improved embryo production and a better embryo quality, very likely as a consequence of a shorter exposure to the free oxygen radicals and the ensuing oxidative stress imposed by overnight culture.

## Introduction

In Vitro Fertilization (IVF) represents the standard, widespread therapeutic approach to human infertility, with more than 8 million babies generated so far around the world^1^. Although IVF procedures are robust, the advent of Intracytoplasmic Sperm Injection^2^ has become the prevalent choice for fertilization in human Assisted Reproduction Technologies (ART). However, IVF is unquestionably a more physiological method of embryo production, for still guarantees a more natural spermatozoa selection for fertilization^3,4^. Moreover, IVF still remains the best option in large animals^5^, where ICSI performs poorly^6^. Therefore, attempts to optimize the classical IVF are welcome. One of the open questions is the definition of the optimal timing of spermatozoa-oocyte co-incubation. Data generated in human ART tend to favour shorter IVF intervals^7^, for longer one’s correlate with an increased polyspermy risk and poor embryo quality. However, the issue is still debated, and some studies have reported contradictory results in term of IVF outcomes and embryo quality^8,9^, as well argued for by the meta-analysis of Zhang and colleagues^10^.

The supporters of short incubation times fear that prolonged gamete co-incubation might expose them to reactive oxygen species (ROS), like superoxide (O_2_^-^), hydroxyl radical (^**.**^OH), singlet oxygen (^1^O_2_), non-radical ones, like hydrogen peroxide (H_2_O_2_) and lipid hydroperoxide (LOOH)^11^. ROS are produced by a variety of biochemical cellular reactions within organelles such as mitochondria, peroxisomes and endoplasmic reticulum^12^. At physiological levels, ROS play important roles in the male fertility because regulate many events related to the acquisition of sperm fertilizing capability, as hyperactivation and phosphorylation involved in sperm capacitation^13^.

However, high ROS free radicals react with numerous biological molecules, such as lipids, proteins and DNA, and can trigger rapid chain reactions causing irreversible cell and DNA damage. Thus, the particular genome conformation in early-stage embryos, very open and highly accessible, might render them vulnerable to ROS, thus compromising further development.

Here, having confirmed and quantified the high levels of ROS produced in IVF medium and finely tracked the early fertilization event, we have observed that restricting gamete co-incubation to only 4 h resulted in a significant amelioration in embryo production outcomes, both quantitatively and qualitatively.

## Results

### Detection of spermatozoa-oocyte interaction

**Figure 1:**
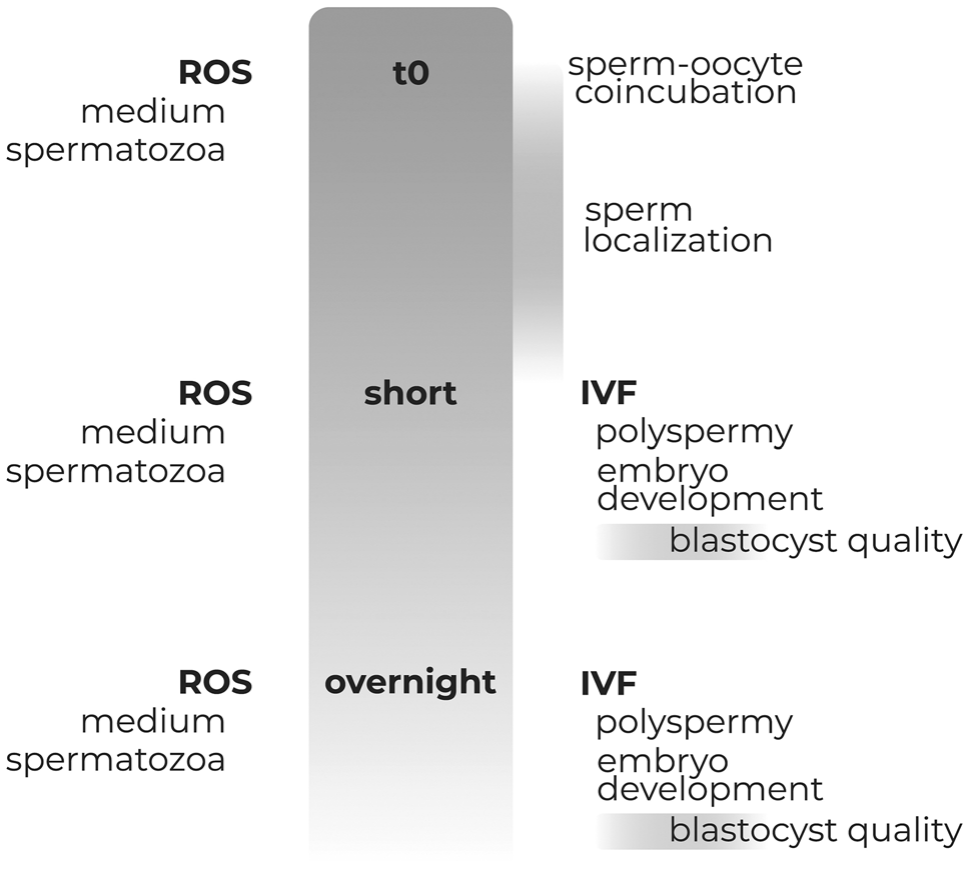
Experimental design. Fertilization window has been detected by localization of sperm within 4 h from sperm-oocyte coincubation. Then, polyspermy, embryo development and blastocysts quality were compared between short and overnight IVF. ROS production from medium only and spermatozoa has been evaluated at t0, after short and overnight incubation. Further details are in the text.

**Figure 2:**
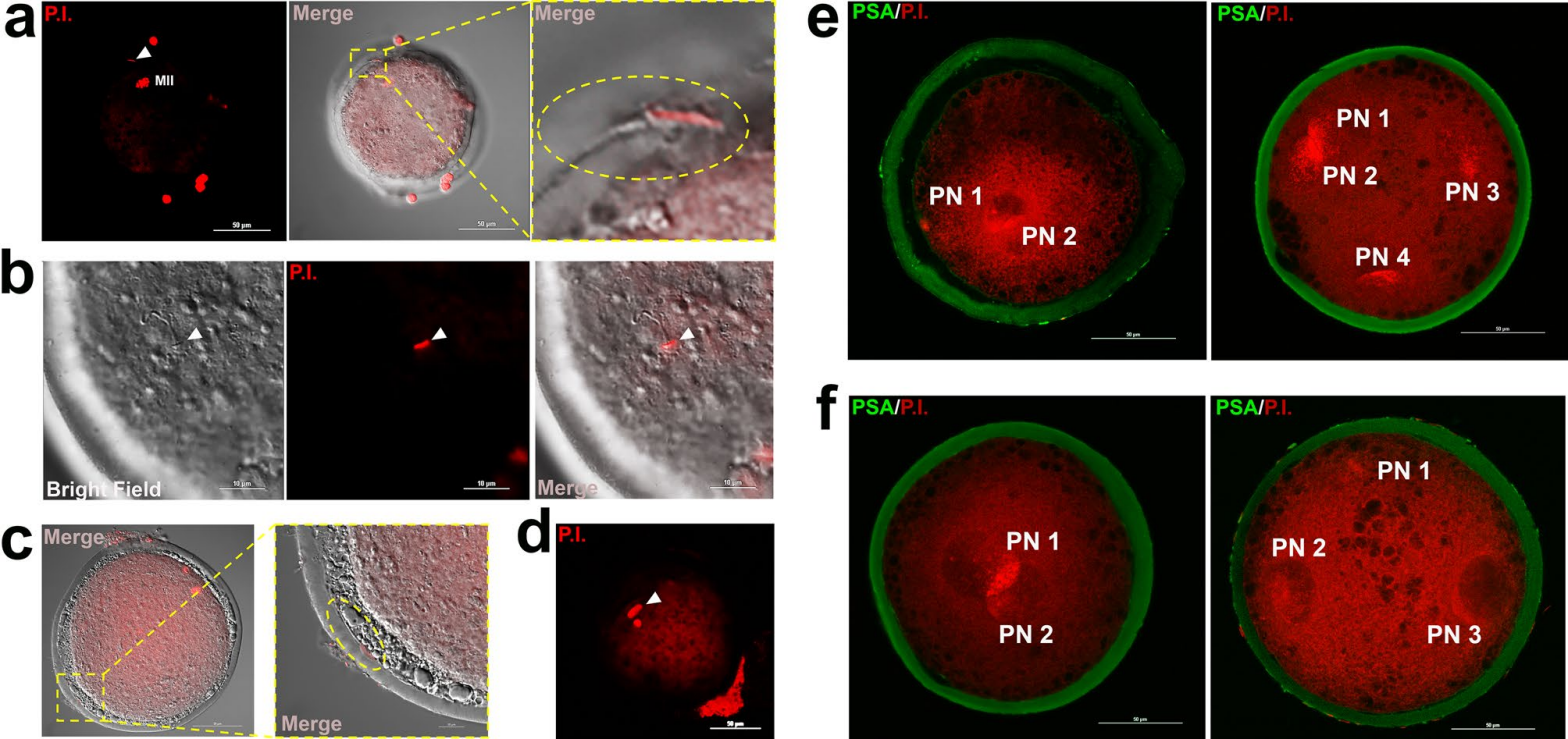
Controlled time-IVF. (**a**) spermatozoon is passing through the zona pellucida (arrow head) 90 min post the beginning of IVF. (**b**) a spermatozoon (arrow head) position at 120 min post IVF. (**c**) Spermatozoon (arrow head) at the perivitelline space at 180 min post-IVF. (**d**) early sperm nucleus decondensation at 240 min after-IVF. (**e**,**f**) Pronuclear evaluation at 14 h post-IVF. (**e**) o/nIVF embryos, on the left normal 2 Pronuclear (2PN) fertilization, on the right polyspermic fertilization (> 2PN). (**f**) shIVF group, on the left normal 2 Pronuclear (2PN) fertilization, on the right polyspermic fertilization (> 2PN). Red signal: Propidium Iodide (P.I.); green signal: Pisum Sativum Agglutinin (PSA). Scale bar in (**a**,**d**,**e**,**f**): 50 μm. Scale bar in (**b**,**c** right): 10 μm.

Matured oocytes were co-incubated with capacitated spermatozoa in IVF medium, and aliquots of oocytes (batches of 7 oocytes sampled every 30 min) were monitored for spermatozoa-oocyte interaction (Fig. 1), previous enzymatic/mechanical cumulus cells removal. The use of Propidium Iodide (PI) allowed us to localize the spermatozoa adhered to, or crossing the ZP, by scanning the entire Z axes in the confocal microscope of individual oocytes. The first spermatozoa bound to ZP were observed 90 min after IVF (54.5%, 6/11) (Fig. 2a), with almost half of the oocyte having ZP-bound spermatozoa by 120 min (45.5%, 5/11) (Fig. 2b). At 180 min, spermatozoa were found in the perivitelline space in the 55.6% of oocytes (5/9) (Fig. 2c). Oocytes monitored 240 min past IVF displayed already a single spermatozoon (66.7%, 4/6), or in fewer cases 2, within the oocyte’s cytoplasm (Fig. 2d). This observation, consistently made in 15 replicates, let to us conclude that 4 h of gamete interaction suffices for successful fertilization (Fig. 1). Based on these findings, we next compared fertilization, polyspermy and embryo development potential between short-IVF (shIVF, 4 h co-incubation) and overnight-IVF (o/nIVF, approximately 16 h of co-incubation).

### Short sperm-eggs co-incubation time reduces polyspermic fertilization

Scoring for polyspermy was carried out at 14 h since gamete co-incubation by observing pronuclear formation. Normal fertilization, 2 ProNuclei (2PN), was preferably observed in shIVF (77%, 94/122) rather than in o/n IVF embryos (60.7%, 68/112). As expected, polyspermic fertilization was significantly increased in o/nIVF group, comparing with shIVF [17.8% (20/112) vs 6.5% (8/122), *P* < 0.05] (Table 1). Representative images of 2PN and polyspermic oocytes after IVF are depicted in (Fig. 2e,f).

**Table 1:** Polyspermy rate.

Sperm-eggs time coincubation	No. oocytes	Non-fertilized (MII) (%)	Fertilized (1PN + 2PN) (%)	1PN (%)	2PN (%)	> 2PN (%)
shIVF	122	12/122 (9.8)	110/122 (90.2)	8/122 (6.5)	94/122 (77.0)	8/122 (6.5)^a^
o/nIVF	112	10/112 (8.9)	102/112 (91.2)	14/112 (12.5)	68/112 (60.7)	20/112 (17.8)^a^

Pronuclei were evaluated after 14 h from IVF starting. Polyspermic zygotes (> 2PN) were higher in o/nIVF than shIVF group. Oocytes with at least one visible pronucleus were considered as fertilized (1PN + 2PN). Values with similar superscript differs significantly (*P* < 0.05).

### Reduced gamete interaction ameliorates embryo development

**Figure 3:**
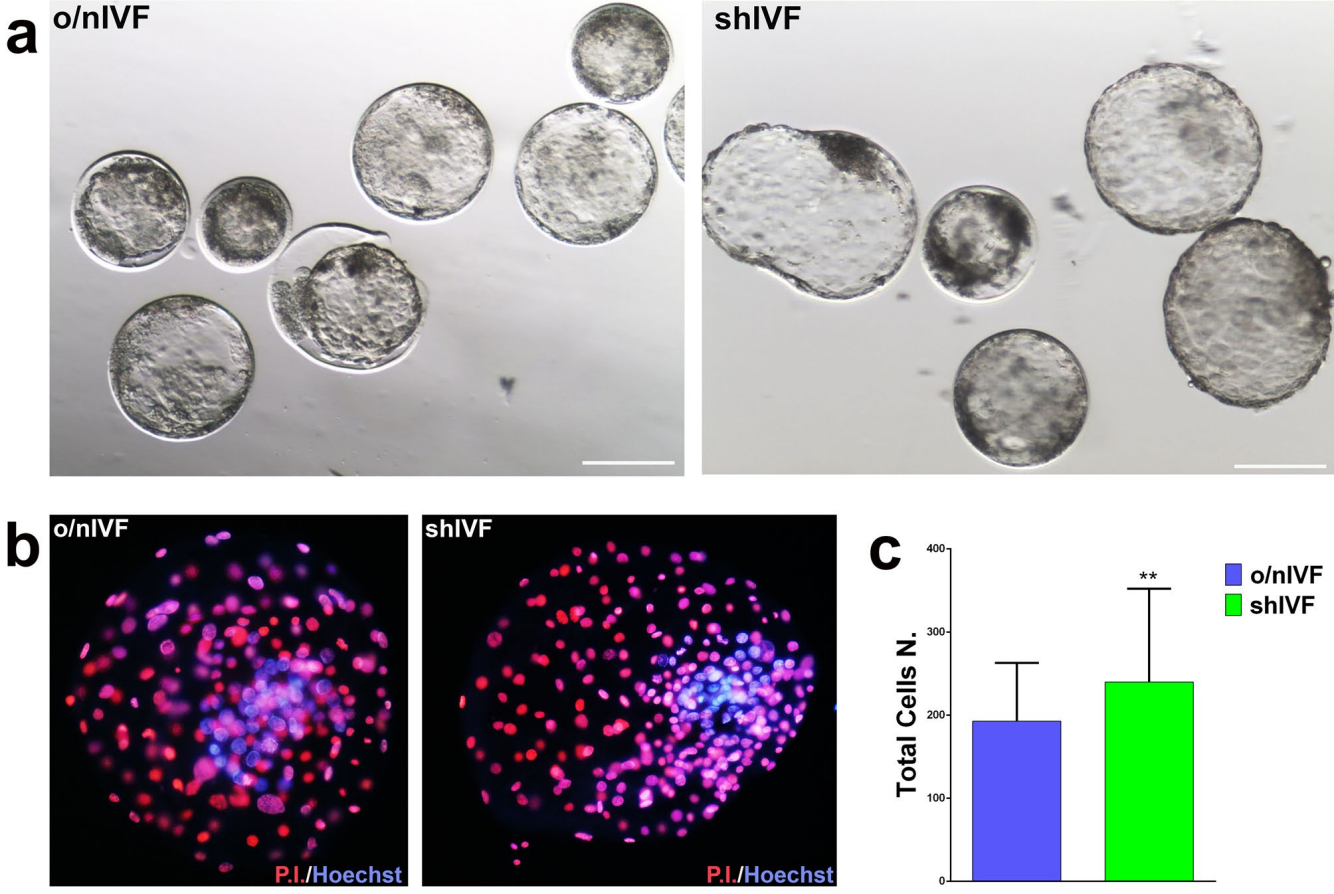
Embryonic development and blastocyst’s cells counting. (**a**) Expanded blastocyst at day 7 of embryo culture produced with convention overnight IVF (on the left) and short IVF (on the right). Scale bar represents: 150 µm. (**b**) Differential staining for blastocyst’s cells counting. On the left, o/nIVF blastocyst, on the right shIVF blastocyst. Chromatin was stained with Hoechst 33,342 (in blue) and Propidium Iodide (in red). (**c**) Graphic representation of blastocyst total cells number. For each comparison a total of 16 blastocysts for o/nIVF and 14 blastocysts for shIVF were evaluated. ** means *P* = 0.0062.

IVF outcomes in both groups are indicated in Table 2. The first cleavage was significantly reduced in o/nIVF, comparing to shIVF [34.6% (81/234) vs 50.6% (122/241) respectively, *P* < 0.001)]. The positive trend continued till the blastocyst stage a day 7, with a higher proportion of blastocysts in shIVF, comparing with o/nIVF [23.5% (55/234) and 29% (70/241) o/nIVF vs shIVF respectively] (Table 2 and Fig. 3a). The beneficial effects of reduced gamete interaction on embryo development were also qualitative.

**Table 2:** Embryo development outcomes.

Sperm-eggs time coincubation	No. oocytes	2-Cells (%)	Expanded blastocysts (%)
shIVF	248	122/241 (50.6)^a^	70/241 (29)
o/nIVF	251	81/234 (34.6)^a^	55/234 (23.5)

Short IVF group showed significantly higher percentage of two-cells stage embryos and lower rate of oocytes not cleaved than overnight IVF group were higher in compared to overnight IVF. Values with similar superscript differs significantly (*P* < 0.05).

### ShIVF positively affects embryo quality

The Total Cell Number (TCN) of the blastocysts was evaluated. Blastocyst at day 7 of shIVF group showed higher TCN, rather than o/nIVF embryos (239.6 ± 42.45 vs 192.5 ± 24.73, respectively; *P* = 0.0062) (Fig. 3b,c).

**Figure 4:**
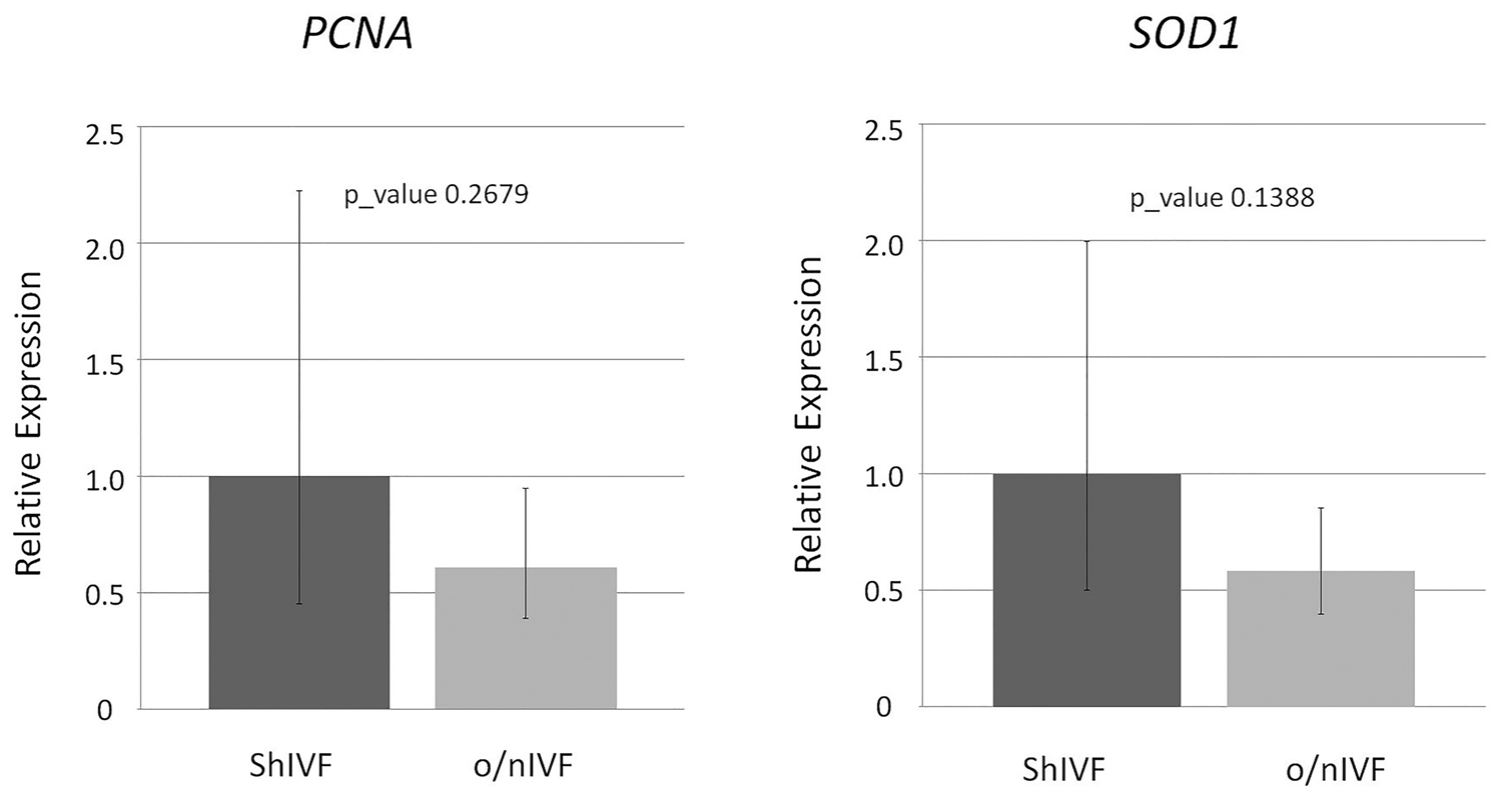
Analysis of molecular markers. The relative expression of the Proliferating Cells Nuclear Antigen (PCNA) and the antioxidant gene (SOD1) were investigated in shIVF and o/nIVF groups (n = 3 each) by RT-qPCR. ACTB were used as reference genes. Gene relative expressions and P-Value (t-test) between shIVF and o/nIVF groups were calculated by the PCR R package.

To get a functional glimpse on the obtained blastocysts, the expression level of a panel of genes (Fig. 4) correlated to embryo development were quantified by reverse transcription quantitative real-time PCR (RT-qPCR). To justify the higher number of cells recorded in the shIVF, we decided to detect the expression of the Proliferating Cells Nuclear Antigen (PCNA) and an antioxidant-related gene, superoxide dismutase (SOD1). Although the two groups (n = 3) did not show significant variation in the relative expression levels of mRNA transcripts for all the genes tested, PCNA and SOD1 showed a decrease in o/nIVF.

### ROS generation is IVF time-dependent

**Figure 5:**
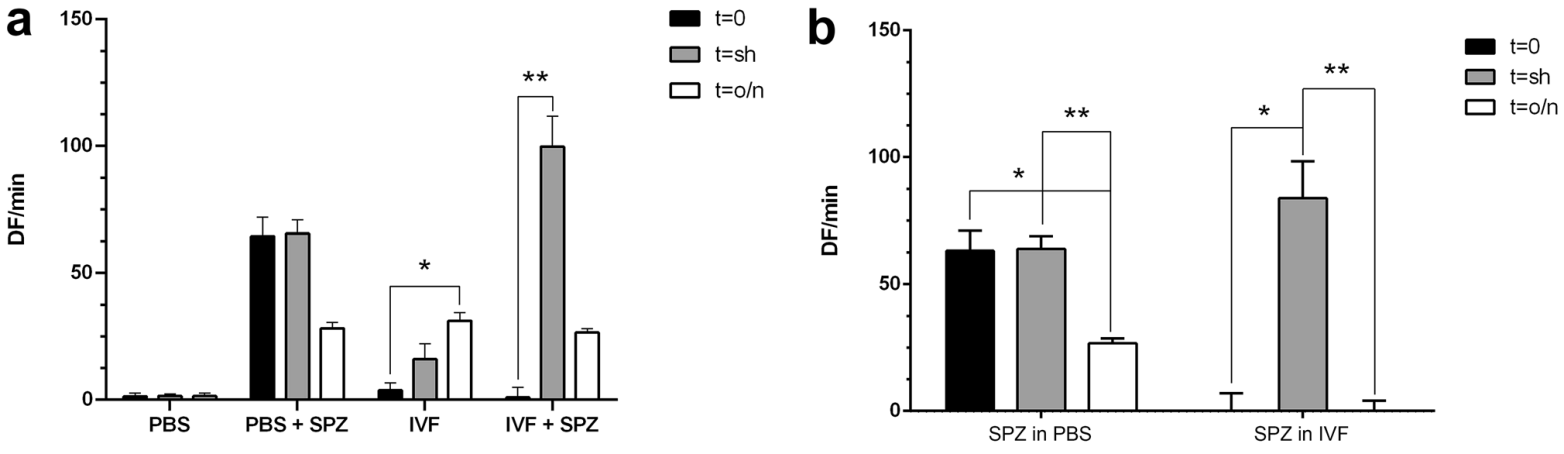
Spermatozoa ROS production. Reactive oxygen species (ROS) generation measured as 2′,7′-dichlorodihydrofluorescein diacetate (H_2_DCFDA) oxidation rate, calculated as mean and standard deviation of fluorescence intensity slope as a function of time (DF/min) in the linear region (corresponding to the first 10 min of the kinetic measurement. (**a**) ROS level detected in PBS and IVF medium with and without spermatozoa in time points t = 0, t = sh (shIVF), t = o/n (o/nIVF). (**b**) ROS level produced by spermatozoa in PBS and IVF medium in time points t = 0, t = sh (shIVF), t = o/n (o/nIVF). * means *P* < 0.005.

Reactive oxygen species (ROS) generation was measured as the oxidation rate of the fluorescent dye 5‐(and 6‐) chloromethyl‐2′,7′-dichlorodihydrofluorescein diacetate (CM-H_2_DCFDA), that is sensitive to a wide range of ROS (H_2_O_2_, ONOO-, superoxide anion, and hydroxyl radicals). The results are expressed as mean and standard deviation of fluorescence intensity slope as a function of time (DF/min) in the linear region (corresponding to the first 10 min of the kinetic measurement) (Fig. 5). To provide an index of oxidation during IVF, ROS production was evaluated in the IVF medium at the beginning (t = 0), and after shIVF time (t = sh) and o/nIVF incubation (t = o/n). To have an index of ROS production from spermatozoa only, we performed the same analysis in PBS.

In the measurements performed on IVF the DF/min is higher at t = o/n than t = 0 (31.17 ± 3.26 VS 3.72 ± 2.98, *P* = 0.0036 respectively) (Fig. 5a). In the coincubation of IVF and spermatozoa the higher DF/min was detected at t = sh (99.80 ± 12.02) respect t = 0 (0.91 ± 4.07), *P* = 0.0036 (Fig. 5a).

Finally, to detect the ROS contribution in PBS and IVF, in both conditions, the DF/min produced in medium + spermatozoa was detracted to only medium revelation. ROS produced by spermatozoa in PBS was lower in t = o/n (26.72 ± 1.87) respect to t = 0 (63.21 ± 7.76) and t = sh (63.90 ± 5.04), *P* = 0.0005 (Fig. 5b). The ROS produced by spermatozoa in IVF medium was higher at t = sh (83.80 ± 14.56) than t = 0 (0 ± 7.03) and t = o/n (0 ± 4.09), *P* = 0.0005 (Fig. 5b).

## Discussion

The current practice in laboratory production of sheep embryos foresees an overnight incubation of mature metaphase II oocytes with capacitated spermatozoa. These settings though comply more with logistic-personnel planning, rather than real biological needs. It has been previously demonstrated that a prolonged permanence of thousands of spermatozoa with the oocytes increases polyspermic fertilization^8,14^. Moreover, the presence of thousands of decaying spermatozoa in a small volume of medium exposes the oocytes to high concentration of lytic, acrosomal enzymes and ROS, a situation that drifts considerably from the physiological situation taking place in the oviduct.

In our work, we have first finely monitored the dynamics of oocyte-spermatozoa interaction leading to fertilization, and have established that 4 h is sufficient to achieve an acceptable fertilization rate (77%). This information prompted us to compare embryo production outcomes using a controlled oocyte-spermatozoa interaction, with the standard overnight procedure. The beneficial effects were evident since the first cleavage, significantly higher in the shIVF group, because an important proportion of embryos of the o/nIVF did not enter the first mitosis (more than 60%).

Recently, it has been reported that the IVF media itself could produce a certain amount of ROS within few hours from the incubation^15^. To investigate the source of ROS in IVF system, we measured the level of ROS produced from IVF medium alone as well as those produced by spermatozoa suspended in IVF medium, from the beginning of IVF up to the end of an overnight incubation. We used the fluorescent dye CM-H_2_DCFDA, sensitive to some ROS, that gives an index of general ROS production from media and cells.

Interestingly, we found a peak of oxidation rate at 4 h (shIVF) from sperm incubation in IVF medium, followed by a strong decrease of oxidation rate after an overnight incubation. This behaviour positively correlates with a higher cleavage rate observed after shIVF and could be likely due to the acquisition of capacitation and fertilization capability of spermatozoa, and the ROS production in this early time of IVF, also reflected by the higher number of spermatozoa still attached to the Zona Pellucida at 4 h from IVF. In fact, it was observed, that ROS inducing lipid peroxidation of sperm membrane enhances the binding of spermatozoa to the ZP^16^, a fundamental aspect of sperm-oocytes interaction. Furthermore, at the end of the canonical o/nIVF, there is a strong decrease in the motility of the spermatozoa, this could explain the low levels of ROS in this group.

Polyspermic fertilization shrank dramatically in the shIVF group, thus confirming previous reports^7,17,18^. The removal of the oocytes from IVF shortly after fertilization positively affected embryonic development, by subtracting them to the high levels of ROS, whose detrimental effects are well established. The positive trend in shIVF group continued till day 7th of culture, with a significantly improvement of the blastocyst development comparing to o/nIVF group (29% vs 23% of total incubated oocytes). Avoiding a long ROS exposure ameliorated not only the number of blastocysts, but even their quality.

Accordingly, we have detected an enhanced expression of SOD in our shIVF blastocysts, as already reported in bovine embryos^19,20^. Likewise, the mitochondrial form of superoxide dismutase, the manganese-SOD (Mn-SOD), has been found to be significantly higher in bovine blastocysts cultured in vivo compared to those cultured in vitro, thus positively associated with a high-quality blastocyst^21^. Besides the morphological appearance, even the total number of cells was significantly higher in shIVF derived blastocysts. The increased proliferation rate was backed up by the upregulation of the PCNA, a specific marker of S-phase of the cell cycle which correlates with cell proliferation and DNA synthesis^22,23^, detected by us in shIVF blastocyst. The upregulated expression of PCNA has been found in ovine embryos produced in vitro at low oxygen concentration, demonstrating that low ROS level induce PCNA up-regulation^24^.

To summarize, our findings demonstrated that a long exposure high ROS level negatively affects embryo development since the early stages. Thus, the final message that our article conveys is that restricting the gametes interaction to an optimal time frame compatible with a satisfactory fertilization rate, probably to be finely investigated in each animal model, quantitatively and qualitatively improves in vitro embryo production in sheep.

## Material and methods

All materials were purchased from Sigma Aldrich, Milan, unless otherwise stated.

### Experimental design

A total of 733 in vitro matured sheep oocytes were co-incubated with spermatozoa in IVF medium. Then, small batches (n = 21; subdivided in 3 replicates) of oocytes were collected every 30 min to check for the presence of a fertilizing spermatozoon. Once the fertilization window has been detected, we performed IVF for a short (shIVF) and conventional overnight (o/nIVF) incubation of spermatozoa-oocytes. IVF outcomes were compared between the two groups for embryo development, polyspermy, blastocysts quality. Furthermore, we evaluated ROS production in IVF medium at time 0 (t0), and after short and overnight incubation (Fig. 1).

### Oocyte recovery and in vitro maturation (IVM)

In vitro maturation was performed as previously described^25^. Briefly, sheep ovaries were collected from local slaughterhouses and transferred to our laboratory at 37 °C within 1 h. Oocytes were aspirated with 21 G needles in HEPES-buffered TCM-199 medium (Gibco, Life Technologies, Milan, Italy) supplemented with 0.005% (w:v) heparin. Cumulus-Oocyte Complexes (COCs) were cultures in 4 wells-dishes containing 0.5 ml IVM medium, composed by bicarbonate-buffered TCM-199 (Gibco) containing 2 mM glutamine, 0.3 mM sodium pyruvate, 100 μM cysteamine, 10% (v:v) fetal bovine serum (FBS) (Gibco), 5 μg/ml follicle stimulating hormone (FSH) (Ovagen, ICP, Auckland, New Zealand), 5 μg/ml luteinizing hormone (LH) and 1 μg/ml β-estradiol, in a humidified atmosphere at 38.5 °C and 5% CO_2_ in air for 24 h. All the process (from oocyte pick-up to the start of IVM) it ends within 1.5 h.

### In vitro fertilization (IVF)

Twenty-four hours post IVM, COCs were observed under the stereomicroscope and only the COCs that presented an expansion of cumulus cells were selected for IVF. After that, COCS were quickly pipetted in 300 U/ml hyaluronidase solution (dissolved in TCM-199), washed twice in H199 and placed into 50 μl drops (in number of 8 to 10 oocytes/drop) of IVF medium (SOF- with 20% oestrus sheep serum and 16 mM isoproterenol), covered by mineral oil. Single straw of frozen semen, containing 100 × 10^6^ spermatozoa, was fast-thawed in 35 °C water and centrifuged in sperm-wash medium (bicarbonate-buffered synthetic oviductal fluid (SOF-) with 0.4% (w:v) fatty-acid free BSA), at 1200 rpm for 5 min. Supernatant was discarded and 5 × 10^6^ spermatozoa were added to each drop and incubated in a humidified atmosphere at 38.5 °C, 5% CO_2_, and 7% O_2_.

### Detection of sperm entrance

To detect the timing of sperm entrance into the oocyte, COCs were first incubated with spermatozoa in IVF medium, then collected (sub batches of 7 oocytes) every 30 min, up to 4 h post IVF, and accurately washed by pipetting in SOF- enriched with 2% (v:v) basal medium Eagle essential amino acids (EAA), 1% (v:v) minimum essential medium (MEM)-nonessential amino acids (NEAA) (Gibco), 1 mM glutamine, and 8 mg/ml fatty acid-free BSA (SOF-aa). Denuded oocytes were fixed in 4% paraformaldehyde (PFA) for 20 min at Room Temperature (RT), washed twice in 0.4% PVP in PBS and stained with 40 μg/ml PSA-FITC (*pisum sativum agglutinin* FITC-conjugated) and 5 μg/ml Propidium Iodide (PI), 8 min in the dark. Finally, oocytes were washed twice in 0.4% PVP in PBS, mounted on the slides and observed under the confocal (Nikon Eclipse Ti-E, Software: NIS-Elements Confocal software) microscope.

### Polyspermy evaluation

To evaluate the polyspermy rate in shIVF and o/nIVF groups, presumptive zygotes were assessed for pronuclear observation after short and overnight incubation with spermatozoa. At 14 h post IVF, both groups of presumptive zygotes were fixed in 4% paraformaldehyde for 20 min at RT, washed twice in 0.4% PVP in PBS, and stained with 40 μg/ml PSA-FITC and 5 μg/ml PI, 8 min in the dark at RT. Thus, presumptive zygotes were washed twice in 0.4% PVP in PBS, mounted on the slides and observed under the confocal microscope (Nikon Eclipse Ti-E, Software: NIS-Elements Confocal software).

### Embryo development

Presumptive zygotes from both groups were cultured in 20 μl drops of SOF-aa covered by mineral oil, in a humidified atmosphere at 38.5 °C, 5% CO_2_, and 7% O_2_. The medium was renewed at day 3 (SOF + aa) (SOF-aa supplemented with 0.27 mg/ml glucose (SOF +), 2% EAA, 1% NEAA), on day 5 (SOF + with 10% of charcoal stripped FBS (csFBS), 2% EAA, 1% NEAA) and on day 6 (1:1 MEM/M199 enriched with 10% cs-FBS, 2.5 μg/ml gentamicin and 1% sodium pyruvate). Embryo development was evaluated at 24 h for 2-cells stage and at 8th day of culture, for blastocyst stage development. Embryo observations and images were captured with Nikon Eclipse Ti2-U inverted microscope using Octax EyeWare Imaging Software (version 2.3.0.372).

### Embryo quality evaluation

#### Blastocyst cell counting

Blastocysts obtained from shIVF and o/nIVF were incubated in Hepes buffered SOF^+^ aa with 1% Triton X-100 and 100 mg/ml *propidium iodide*, for 7 s at room temperature, then transferred in 100% ethanol with 25 mg/ml bisbenzimide Hoechst 33,258, 3 h at 4 °C, mounted with glycerol and observed under confocal microscope Nikon Eclipse Ti-E and analyzed for cell counting using G2A and UV filter. All blastocysts were analyzed at 7th day of culture. For each blastocyst, total cell number (TCN) was compared between groups.

#### RT-qPCR

Total RNA was extracted from blastocyst (n = 3) in shIVF and o/nIVF groups (n = 3) using TRIzol (Invitrogen, Carlsbad, CA) and purified with NucleoSpin miRNA kit (Macherey–Nagel, Germany), following the protocol in combination with TRIzol lysis with small and large RNA in one fraction (total RNA) with minor modification. Briefly, 150 μl of TRIzol were added into each pool of three blastocysts, homogenized and incubated 5 min at room temperature. The mixture was then added 30 μl of chloroform, homogenized and incubated 3 min at room temperature. Aqueous phase containing RNA was separated after centrifugation (15,000*g*, 12 min at 4 °C) and added 187.5 μl of MX buffer. The mixture was purified with the NucleoSpin RNA column following manufactured instruction. RNA was eluted with 12 μl of RNAse free water and directly reverse transcribed into cDNA in a total reaction volume of 18 μl. 10 μl of RNA was added of 0.25 μl of random hexamers, 0.25 μl oligodT, 0.5 μl dNTPs and incubated at 65 °C for 5 min and placed at 4 °C. Mixture was then added of 4 μl RT buffer (5 x), 1 μl of DTT (0.1 M), 1 μl RNase inhibitor, and 1 μl of SuperScript II Reverse Transcriptase (Thermo Fisher, Waltham, MA USA). Reverse transcription was carried out at 25 °C for 5 min, 42 °C for 1 h and 70 °C for 15 min.

The transcript level of the different genes, *PCNA, SOD1* and *ACTB* as a reference gene, were measured by RT-qPCR. Table 3 reported the previous reported primers used in this study. Primers were designed from specific exon-exon junctions to avoid amplifying genomic DNA. RT-qPCR was performed with three technical replicate each, using 4 μl of diluted cDNA (1:20 Vol.), 5 μl of the Power SYBR Green Master Mix (Applied Biosystems, Carlsbad, California, USA) and 0.5 μl of forward and reverse primers (final concentration 600 nM for PCNA and ACTB and 900 nM for SOD1) with QuantStudio 6 Flex Real-Time PCR Systems (Applied Biosystems, Carlsbad, California, USA). RT-QPCR efficiencies calculated from slope were 1.14, 1.16 and 0.94 for *PCNA, SOD1* and *ACTB,* respectively. Gene relative expressions between shIVF and o/nIVF groups and P-Value (t-test) were calculated by the PCR R package^26^.

**Table 3:** Gene analyzed.

Gene	Primer sequences (5ˊ–3ˊ)	Lenght of PCR product	Tm ( °C)	Gene position
*PCNA*	F:AGCCACTCCACTGTCTCCTACA	123	60	F exon 5; R in exon 6
	R:TCATCCTCGATCTTGGGAGCC			
*SOD1*	F:CACTTCGAGGCAAAGGGAGA	167	60	F exon 1 R in exon 3
	R:CCTTTGGCCCACCGTGTTTT			
*ACTB*	F:CCATCGGCAATGAGCGGT	146	60	F exon 4 R in exon5
	R:CGTGTTGGCGTAGAGGTC			

Previous reported primers used in this study. Reference for gene: *PCNA*^27^, *SOD1*^27^, *ACTB*^28^.

### Measurement of ROS generation

The amount of ROS produced by the IVF medium and spermatozoa has been calculated by the oxidation rate of the fluorescent probe CM-H_2_DCFDA. To estimate the rate of ROS produced by spermatozoa, we incubated spermatozoa both in IVF medium and in PBS. Measurements were taken at three temporal stages: at the beginning (0 h), at short (shIVF) and overnight (o/nIVF) incubation. To avoid photo-bleaching, IVF medium was diluted 1:9 with PBS, immediately before measurement^15^. For each group, a total of 200 μl of cell-free or spermatozoa-containing medium/buffer, were placed in each well of a 96-well plate, and then mixed with CM-H_2_DCFDA dye to a final dye working concentration of 3 μM.

Fluorescence emission measurements were carried out in a multimode ENSPIRE plate reader (Perkin Elmer) at a constant temperature of 38 °C. To avoid condensation, a difference of 0.3 °C between the bottom and the top of the plate was set. The excitation and emission wavelengths were set respectively to 490 nm and 520 nm. 40 repeats were measured for each well with an interval of 1 min for each repeat. For each repeat, 100 flashes were lighted at a height of 8 mm from the top of the plate.

The kinetics of CM-H_2_DCFDA oxidation rate was evaluated from the slope of the fluorescence emission intensity in the initial 10 min of the total recording time. Results are reported as mean ± SD of triplicate measurements.

A calibration curve was set-up by measuring the oxidation rate of the dye as a function of H_2_O_2_ concentration in PBS. Dye-free PBS/IVF medium have been used as negative controls.

### Statistical analysis

Fisher’s exact test was used to compare 2PN and in vitro embryo development outcomes. The comparison of blastocyst total cells numbers was handled in GraphPad Prism for Windows (Version 6.01, GraphPad software, CA, USA) using the Wilcoxon non-parametric t-test. Test was based on 16 expanded blastocysts for o/nIVF group and 14 blastocysts for shIVF. Data are reported as means ± standard error mean (SEM). For the ROS rate was performed ANOVA non-parametric test (Kruskal–Wallis test), data were reported as Mean ± SD. For all the tests the level of significance has been set at *P* < 0.05.
